# Butyl 2-(5-bromo-3-methyl­sulfinyl-1-benzofuran-2-yl)acetate

**DOI:** 10.1107/S1600536808043985

**Published:** 2009-01-08

**Authors:** Hong Dae Choi, Pil Ja Seo, Byeng Wha Son, Uk Lee

**Affiliations:** a Department of Chemistry, Dongeui University, San 24 Kaya-dong Busanjin-gu, Busan 614-714, Republic of Korea; b Department of Chemistry, Pukyong National University, 599-1 Daeyeon 3-dong Nam-gu, Busan 608-737, Republic of Korea

## Abstract

In the title compound, C_15_H_17_BrO_4_S, the methyl­sulfinyl O atom and the methyl substituents lie on opposite sides of the plane through the benzofuran fragment. The crystal structure is stabilized by π–π inter­actions between the benzene rings of neighbouring mol­ecules [centroid–centroid distance = 3.698 (4) Å], and by C—H⋯π inter­actions between a methyl­ene H atom of the butyl group and the benzene ring of the benzofuran system. Additionally, the crystal structure exhibits weak inter­molecular C—H⋯O contacts. The butyl group is disordered over two positions, with site-occupancy factors, from refinement, of 0.720 (8) and 0.280 (8).

## Related literature

For the crystal structures of similar alkyl 2-(5-bromo-3-methyl­sulfinyl-1-benzofuran-2-yl)acetate derivatives. see: Choi *et al.* (2008*a* ▶,*b* ▶).
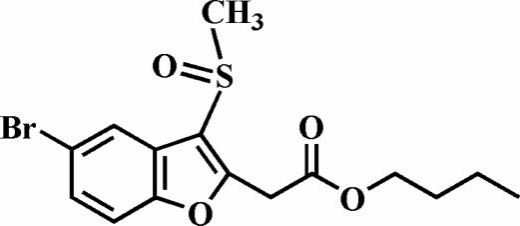


## Experimental

### 

#### Crystal data

C_15_H_17_BrO_4_S
*M* *_r_* = 373.26
Triclinic, 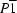

*a* = 8.420 (1) Å
*b* = 10.255 (1) Å
*c* = 10.306 (1) Å
α = 97.503 (2)°
β = 99.711 (2)°
γ = 108.678 (2)°
*V* = 814.55 (15) Å^3^
*Z* = 2
Mo *K*α radiation
μ = 2.66 mm^−1^
*T* = 298 (2) K
0.40 × 0.40 × 0.30 mm

#### Data collection

Bruker SMART CCD diffractometer
Absorption correction: multi-scan (*SADABS*; Sheldrick, 1999 ▶) *T*_min_ = 0.353, *T*_max_ = 0.451
6560 measured reflections
3179 independent reflections
2645 reflections with *I* > 2σ(*I*)
*R*_int_ = 0.017

#### Refinement

*R*[*F* ^2^ > 2σ(*F* ^2^)] = 0.033
*wR*(*F* ^2^) = 0.089
*S* = 1.14
3179 reflections
229 parameters
64 restraints
H-atom parameters constrained
Δρ_max_ = 0.30 e Å^−3^
Δρ_min_ = −0.50 e Å^−3^

### 

Data collection: *SMART* (Bruker, 2001 ▶); cell refinement: *SAINT* (Bruker, 2001 ▶); data reduction: *SAINT*; program(s) used to solve structure: *SHELXS97* (Sheldrick, 2008 ▶); program(s) used to refine structure: *SHELXL97* (Sheldrick, 2008 ▶); molecular graphics: *ORTEP-3* (Farrugia, 1997 ▶) and *DIAMOND* (Brandenburg, 1998 ▶); software used to prepare material for publication: *SHELXL97*.

## Supplementary Material

Crystal structure: contains datablocks global, I. DOI: 10.1107/S1600536808043985/tk2347sup1.cif

Structure factors: contains datablocks I. DOI: 10.1107/S1600536808043985/tk2347Isup2.hkl

Additional supplementary materials:  crystallographic information; 3D view; checkCIF report

## Figures and Tables

**Table 1:** Hydrogen-bond geometry (Å, °)

*D*—H⋯*A*	*D*—H	H⋯*A*	*D*⋯*A*	*D*—H⋯*A*
C12*A*—H12*A*⋯*Cg*^i^	0.97	2.78	3.698 (5)	158
C5—H5⋯O3^ii^	0.93	2.55	3.405 (3)	153
C9—H9*B*⋯O4^iii^	0.97	2.30	3.248 (3)	167

Symmetry codes: (i) 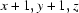; (ii) 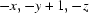; (iii) 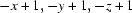.

**Fig. 1.**
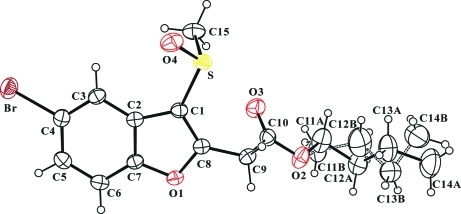
The molecular structure of (I), showing displacement ellipsoids drawn at the 30% probability level. The butyl group is disordered over two positions with the major component having a site occupancy = 0.720 (8).

**Fig. 2.**
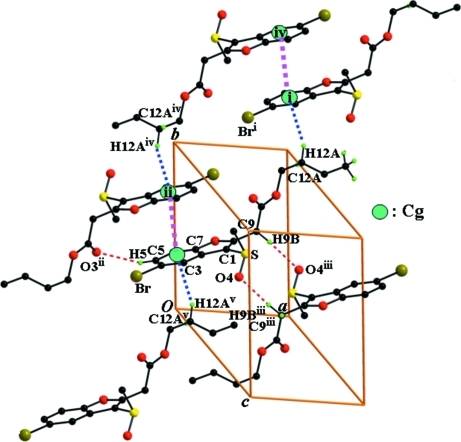
Diagram illustrating the π—π, C—H···π and C—H···O interactions (dotted lines) in the crystal structure of (I). Cg denotes a ring centroid. The disordered component of the butyl group, part B, has been omitted for clarity as have H atoms not involved in intermolecular contacts. Symmetry codes: (i) x + 1, y + 1, z; (ii) -x, 1 - y, -z; (iii) -x + 1, -y + 1, -z + 1; (iv) -x + 1, -y + 2, -z; (v) x - 1, y - 1, z.

## supplementary crystallographic information

### Comment

This work is related to our previous communications on the synthesis and structure of alkyl 2-(5-bromo-3-methylsulfinyl-1-benzofuran-2-yl)acetate analogues, *viz*. isopropyl 2-(5-bromo-3-methylsulfinyl-1-benzofuran-2-yl)acetate (Choi *et al.*, 2008*a*) and methyl 2-(5-bromo-3-methylsulfinyl-1-benzofuran-2-yl)acetate (Choi *et al.*, 2008*b*). Herein, we describe the crystal structure of the title compound, (I).

The benzofuran unit is essentially planar, with a mean deviation of 0.012 (2) Å from the least-squares plane defined by the nine constituent atoms. The butyl group is disordered over two positions with site-occupancy factors of 0.720 (8) (for atoms labelled B) and 0.280 (8) (B) in Fig. 1. The molecular packing is stabilized by intermolecular π—π interactions: the *Cg*···*Cg*^ii^ distance is 3.698 (4) Å, where *Cg* is the centroid of the C2–C7 ring, symmetry code as in Fig. 2. The molecular packing is further stabilized by C—H···π interactions between the methylene-H and the benzene ring of the benzofuran system, with a C12A—H12A···*Cg*^i^ separation of 2.78 Å, Table 1; *Cg* is the centroid of the C2–C7 benzene ring. In addition, weak intermolecular C—H···O contacts are observed, Table 1. One C-H···O contact occurs between a benzene-H and the O3-oxygen, and a second between a methylene-H and the O4-oxygen atom.

### Experimental

77% 3-Chloroperoxybenzoic acid (148 mg, 0.66 mmol) was added in small portions to a stirred solution of butyl 2-(5-bromo-3-methylsulfanyl-1-benzofuran-2-yl)acetate (214 mg, 0.6 mmol) in dichloromethane (30 ml) at 273 K. After being stirred for 3 h at room temperature, the mixture was washed with saturated sodium bicarbonate solution and the organic layer separated, dried over magnesium sulfate, filtered and concentrated in vacuum. The residue was purified by column chromatography (hexane-ethyl acetate, 1:2 *v*/*v*) to afford (I) as a colorless solid [yield 80%, m.p. 381–382 K; *R*_f_ = 0.65 (hexane-ethyl acetate, 1;2 *v*/*v*)]. Single crystals were obtained by evaporation of an acetone solution of (I). Spectroscopic analysis: ^1^H NMR (CDCl_3_, 400 MHz) δ 0.92 (t, J = 7.32 Hz, 3H), 1.31–1.41 (m, 2H), 1.59–1.67 (m, 2H), 3.07 (s, 3H), 4.04 (s, 2H), 4.15 (t, J = 6.6 Hz, 2H), 7.39 (d, J = 8.8 Hz, 1H), 7.49 (dd, J = 8.8 Hz and J = 2.2 Hz, 1H), 8.11 (d, J = 1.84 Hz, 1H); EI—MS 374 [*M*+2], 372 [*M*^+^].

### Refinement

All H atoms were geometrically positioned and refined using a riding model, with C—H = 0.93 Å for aryl-, 0.97 Å for methylene-, and 0.96 Å for methyl-H atoms, and with *U*iso(H) = 1.2*U*eq(C) for the aryl- and methylene-H atoms, and 1.5*U*eq(C) for methyl-H atoms. The butyl group was found to be disordered over two positions and modelled with site-occupancy factors, from refinement, of 0.720 (8) (C11A–C14A)) and 0.280 (8) (C11B–C14B). The displacement ellipsoids of part B part were restrained using command ISOR (0.01), both sets of C atoms were restrained using the command DELU, and the C—C distances were restrained to 1.480 (2) Å using command *DFIX*.

### Crystal data

**Table:**

C_15_H_17_BrO_4_S	*Z* = 2
*M**_r_* = 373.26	*F*(000) = 380
Triclinic, *P*1	*D*_x_ = 1.522 Mg m^−^^3^
Hall symbol: -P 1	Mo *K*α radiation, λ = 0.71073 Å
*a* = 8.420 (1) Å	Cell parameters from 3446 reflections
*b* = 10.255 (1) Å	θ = 2.6–27.0°
*c* = 10.306 (1) Å	µ = 2.66 mm^−^^1^
α = 97.503 (2)°	*T* = 298 K
β = 99.711 (2)°	Block, colorless
γ = 108.678 (2)°	0.40 × 0.40 × 0.30 mm
*V* = 814.55 (15) Å^3^	

### Data collection

**Table:**

Bruker SMART CCD diffractometer	3179 independent reflections
Radiation source: fine-focus sealed tube	2645 reflections with *I* > 2σ(*I*)
graphite	*R*_int_ = 0.017
Detector resolution: 10.0 pixels mm^-1^	θ_max_ = 26.0°, θ_min_ = 2.1°
φ and ω scans	*h* = −10→10
Absorption correction: multi-scan (*SADABS*; Sheldrick, 1999)	*k* = −12→12
*T*_min_ = 0.353, *T*_max_ = 0.451	*l* = −12→12
6560 measured reflections	

### Refinement

**Table:**

Refinement on *F*^2^	Primary atom site location: structure-invariant direct methods
Least-squares matrix: full	Secondary atom site location: difference Fourier map
*R*[*F*^2^ > 2σ(*F*^2^)] = 0.033	Hydrogen site location: difference Fourier map
*wR*(*F*^2^) = 0.089	H-atom parameters constrained
*S* = 1.14	*w* = 1/[σ^2^(*F*_o_^2^) + (0.0442*P*)^2^ + 0.2004*P*] where *P* = (*F*_o_^2^ + 2*F*_c_^2^)/3
3179 reflections	(Δ/σ)_max_ < 0.001
229 parameters	Δρ_max_ = 0.30 e Å^−^^3^
64 restraints	Δρ_min_ = −0.50 e Å^−^^3^

### Special details

**Table:**

Geometry. All e.s.d.'s (except the e.s.d. in the dihedral angle between two l.s. planes) are estimated using the full covariance matrix. The cell e.s.d.'s are taken into account individually in the estimation of e.s.d.'s in distances, angles and torsion angles; correlations between e.s.d.'s in cell parameters are only used when they are defined by crystal symmetry. An approximate (isotropic) treatment of cell e.s.d.'s is used for estimating e.s.d.'s involving l.s. planes.
Refinement. Refinement of *F*^2^ against ALL reflections. The weighted *R*-factor *wR* and goodness of fit *S* are based on *F*^2^, conventional *R*-factors *R* are based on *F*, with *F* set to zero for negative *F*^2^. The threshold expression of *F*^2^ > σ(*F*^2^) is used only for calculating *R*-factors(gt) *etc*. and is not relevant to the choice of reflections for refinement. *R*-factors based on *F*^2^ are statistically about twice as large as those based on *F*, and *R*- factors based on ALL data will be even larger.

### Fractional atomic coordinates and isotropic or equivalent isotropic displacement parameters (Å^2^)

**Table:**

	*x*	*y*	*z*	*U*_iso_*/*U*_eq_	Occ. (<1)
Br	−0.42599 (4)	0.24082 (3)	0.12584 (3)	0.06775 (14)	
S	0.32177 (9)	0.58854 (7)	0.45871 (6)	0.04988 (17)	
O1	0.3033 (2)	0.46045 (17)	0.07725 (15)	0.0442 (4)	
O2	0.7594 (3)	0.8276 (2)	0.2120 (2)	0.0686 (6)	
O3	0.5174 (3)	0.8291 (2)	0.2711 (2)	0.0721 (6)	
O4	0.2197 (3)	0.4784 (2)	0.52321 (18)	0.0618 (5)	
C1	0.2665 (3)	0.5195 (2)	0.2849 (2)	0.0413 (5)	
C2	0.0997 (3)	0.4347 (2)	0.2012 (2)	0.0401 (5)	
C3	−0.0682 (3)	0.3882 (3)	0.2186 (2)	0.0447 (5)	
H3	−0.0941	0.4108	0.3010	0.054*	
C4	−0.1944 (3)	0.3070 (3)	0.1078 (3)	0.0472 (6)	
C5	−0.1604 (4)	0.2716 (3)	−0.0175 (3)	0.0509 (6)	
H5	−0.2500	0.2159	−0.0889	0.061*	
C6	0.0052 (3)	0.3188 (3)	−0.0354 (2)	0.0478 (6)	
H6	0.0307	0.2966	−0.1181	0.057*	
C7	0.1318 (3)	0.4008 (2)	0.0749 (2)	0.0414 (5)	
C8	0.3827 (3)	0.5320 (2)	0.2067 (2)	0.0417 (5)	
C9	0.5701 (3)	0.6117 (3)	0.2310 (3)	0.0460 (6)	
H9A	0.6150	0.5767	0.1586	0.055*	
H9B	0.6281	0.5959	0.3140	0.055*	
C10	0.6090 (3)	0.7675 (3)	0.2403 (3)	0.0506 (6)	
C11A	0.8140 (8)	0.9810 (14)	0.2252 (11)	0.087 (3)	0.720 (8)
H11A	0.8117	1.0245	0.3139	0.104*	0.720 (8)
H11B	0.7375	1.0053	0.1590	0.104*	0.720 (8)
C12A	0.9913 (6)	1.0302 (6)	0.2033 (6)	0.0749 (16)	0.720 (8)
H12A	1.0207	1.1232	0.1821	0.090*	0.720 (8)
H12B	1.0036	0.9664	0.1307	0.090*	0.720 (8)
C13A	1.1021 (6)	1.0324 (8)	0.3331 (7)	0.098 (2)	0.720 (8)
H13A	1.0712	1.0833	0.4050	0.117*	0.720 (8)
H13B	1.0766	0.9366	0.3464	0.117*	0.720 (8)
C14A	1.2899 (7)	1.0964 (9)	0.3450 (10)	0.134 (3)	0.720 (8)
H14A	1.3499	1.0967	0.4330	0.202*	0.720 (8)
H14B	1.3173	1.1911	0.3309	0.202*	0.720 (8)
H14C	1.3243	1.0429	0.2788	0.202*	0.720 (8)
C11B	0.806 (2)	0.973 (3)	0.183 (2)	0.070 (5)	0.280 (8)
H11C	0.7231	1.0153	0.2025	0.084*	0.280 (8)
H11D	0.8138	0.9721	0.0903	0.084*	0.280 (8)
C12B	0.9768 (19)	1.049 (2)	0.275 (3)	0.127 (7)	0.280 (8)
H12C	0.9614	1.0361	0.3645	0.153*	0.280 (8)
H12D	1.0003	1.1475	0.2746	0.153*	0.280 (8)
C13B	1.1377 (19)	1.0231 (18)	0.2628 (16)	0.087 (5)	0.280 (8)
H13C	1.1320	0.9270	0.2622	0.105*	0.280 (8)
H13D	1.1884	1.0601	0.1915	0.105*	0.280 (8)
C14B	1.207 (3)	1.117 (2)	0.3964 (16)	0.124 (6)	0.280 (8)
H14D	1.3305	1.1547	0.4133	0.186*	0.280 (8)
H14E	1.1729	1.0655	0.4643	0.186*	0.280 (8)
H14F	1.1634	1.1931	0.3985	0.186*	0.280 (8)
C15	0.2192 (5)	0.7173 (3)	0.4576 (3)	0.0679 (8)	
H15A	0.0971	0.6710	0.4266	0.102*	
H15B	0.2615	0.7788	0.3987	0.102*	
H15C	0.2442	0.7710	0.5468	0.102*	

### Atomic displacement parameters (Å^2^)

**Table:**

	*U*^11^	*U*^22^	*U*^33^	*U*^12^	*U*^13^	*U*^23^
Br	0.04457 (17)	0.0686 (2)	0.0882 (3)	0.01726 (14)	0.01642 (15)	0.01369 (16)
S	0.0507 (4)	0.0625 (4)	0.0376 (3)	0.0229 (3)	0.0092 (3)	0.0067 (3)
O1	0.0471 (9)	0.0509 (10)	0.0400 (9)	0.0212 (8)	0.0158 (7)	0.0093 (7)
O2	0.0515 (11)	0.0466 (11)	0.1135 (17)	0.0178 (9)	0.0256 (11)	0.0246 (11)
O3	0.0637 (13)	0.0590 (12)	0.1004 (16)	0.0315 (11)	0.0233 (12)	0.0072 (11)
O4	0.0721 (13)	0.0801 (14)	0.0466 (10)	0.0348 (11)	0.0218 (9)	0.0265 (9)
C1	0.0462 (13)	0.0449 (13)	0.0361 (12)	0.0191 (10)	0.0103 (10)	0.0099 (10)
C2	0.0474 (13)	0.0399 (12)	0.0393 (12)	0.0203 (10)	0.0129 (10)	0.0123 (10)
C3	0.0474 (13)	0.0471 (13)	0.0471 (14)	0.0220 (11)	0.0160 (11)	0.0146 (11)
C4	0.0448 (13)	0.0421 (13)	0.0588 (15)	0.0192 (11)	0.0119 (11)	0.0135 (11)
C5	0.0537 (15)	0.0468 (14)	0.0504 (14)	0.0221 (12)	0.0019 (12)	0.0044 (11)
C6	0.0561 (15)	0.0515 (14)	0.0400 (13)	0.0261 (12)	0.0099 (11)	0.0061 (11)
C7	0.0449 (13)	0.0420 (12)	0.0440 (12)	0.0211 (10)	0.0134 (10)	0.0117 (10)
C8	0.0462 (13)	0.0441 (13)	0.0399 (12)	0.0209 (10)	0.0111 (10)	0.0112 (10)
C9	0.0447 (13)	0.0512 (14)	0.0480 (14)	0.0215 (11)	0.0144 (11)	0.0132 (11)
C10	0.0463 (14)	0.0517 (15)	0.0531 (15)	0.0191 (12)	0.0068 (11)	0.0085 (12)
C11A	0.072 (4)	0.059 (4)	0.137 (7)	0.029 (3)	0.019 (4)	0.032 (5)
C12A	0.075 (3)	0.044 (2)	0.105 (4)	0.013 (2)	0.022 (3)	0.028 (3)
C13A	0.072 (3)	0.080 (4)	0.117 (5)	0.002 (3)	0.004 (3)	0.024 (4)
C14A	0.077 (4)	0.120 (5)	0.175 (7)	−0.001 (4)	0.024 (4)	0.018 (5)
C11B	0.072 (7)	0.040 (8)	0.096 (9)	0.008 (5)	0.016 (6)	0.034 (7)
C12B	0.110 (8)	0.109 (10)	0.162 (12)	0.054 (8)	−0.005 (7)	0.028 (9)
C13B	0.085 (7)	0.086 (8)	0.089 (8)	0.028 (6)	0.022 (6)	0.012 (6)
C14B	0.122 (10)	0.135 (10)	0.109 (9)	0.053 (8)	0.012 (7)	0.000 (7)
C15	0.087 (2)	0.0654 (19)	0.0637 (18)	0.0394 (17)	0.0291 (16)	0.0087 (15)

### Geometric parameters (Å, °)

**Table:**

Br—C4	1.899 (3)	C11A—H11A	0.9700
S—O4	1.491 (2)	C11A—H11B	0.9700
S—C1	1.762 (2)	C12A—C13A	1.489 (2)
S—C15	1.794 (3)	C12A—H12A	0.9700
O1—C7	1.370 (3)	C12A—H12B	0.9700
O1—C8	1.376 (3)	C13A—C14A	1.482 (2)
O2—C10	1.319 (3)	C13A—H13A	0.9700
O2—C11A	1.471 (14)	C13A—H13B	0.9700
O2—C11B	1.50 (3)	C14A—H14A	0.9600
O3—C10	1.199 (3)	C14A—H14B	0.9600
C1—C8	1.355 (3)	C14A—H14C	0.9600
C1—C2	1.444 (3)	C11B—C12B	1.481 (2)
C2—C3	1.391 (3)	C11B—H11C	0.9700
C2—C7	1.396 (3)	C11B—H11D	0.9700
C3—C4	1.380 (4)	C12B—C13B	1.483 (2)
C3—H3	0.9300	C12B—H12C	0.9700
C4—C5	1.396 (4)	C12B—H12D	0.9700
C5—C6	1.376 (4)	C13B—C14B	1.481 (2)
C5—H5	0.9300	C13B—H13C	0.9700
C6—C7	1.380 (3)	C13B—H13D	0.9700
C6—H6	0.9300	C14B—H14D	0.9600
C8—C9	1.486 (3)	C14B—H14E	0.9600
C9—C10	1.511 (4)	C14B—H14F	0.9600
C9—H9A	0.9700	C15—H15A	0.9600
C9—H9B	0.9700	C15—H15B	0.9600
C11A—C12A	1.482 (2)	C15—H15C	0.9600
			
O4—S—C1	106.92 (12)	C11A—C12A—H12A	110.8
O4—S—C15	105.78 (14)	C13A—C12A—H12A	110.8
C1—S—C15	98.46 (13)	C11A—C12A—H12B	110.8
C7—O1—C8	106.62 (17)	C13A—C12A—H12B	110.8
C10—O2—C11A	115.2 (3)	H12A—C12A—H12B	108.9
C10—O2—C11B	120.0 (10)	C14A—C13A—C12A	115.7 (6)
C11A—O2—C11B	16.2 (11)	C14A—C13A—H13A	108.4
C8—C1—C2	107.4 (2)	C12A—C13A—H13A	108.4
C8—C1—S	123.77 (19)	C14A—C13A—H13B	108.4
C2—C1—S	128.70 (18)	C12A—C13A—H13B	108.4
C3—C2—C7	119.5 (2)	H13A—C13A—H13B	107.4
C3—C2—C1	135.8 (2)	C13A—C14A—H14A	109.5
C7—C2—C1	104.6 (2)	C13A—C14A—H14B	109.5
C4—C3—C2	116.8 (2)	H14A—C14A—H14B	109.5
C4—C3—H3	121.6	C13A—C14A—H14C	109.5
C2—C3—H3	121.6	H14A—C14A—H14C	109.5
C3—C4—C5	123.2 (2)	H14B—C14A—H14C	109.5
C3—C4—Br	118.51 (19)	O2—C11B—C12B	104 (2)
C5—C4—Br	118.29 (19)	O2—C11B—H11C	111.1
C6—C5—C4	120.2 (2)	C12B—C11B—H11C	111.1
C6—C5—H5	119.9	O2—C11B—H11D	111.1
C4—C5—H5	119.9	C12B—C11B—H11D	111.1
C5—C6—C7	116.8 (2)	H11C—C11B—H11D	109.0
C5—C6—H6	121.6	C11B—C12B—C13B	125 (2)
C7—C6—H6	121.6	C11B—C12B—H12C	106.1
O1—C7—C6	125.9 (2)	C13B—C12B—H12C	106.1
O1—C7—C2	110.7 (2)	C11B—C12B—H12D	106.1
C6—C7—C2	123.5 (2)	C13B—C12B—H12D	106.1
C1—C8—O1	110.7 (2)	H12C—C12B—H12D	106.3
C1—C8—C9	133.3 (2)	C14B—C13B—C12B	83.6 (14)
O1—C8—C9	115.9 (2)	C14B—C13B—H13C	114.7
C8—C9—C10	112.3 (2)	C12B—C13B—H13C	114.7
C8—C9—H9A	109.1	C14B—C13B—H13D	114.7
C10—C9—H9A	109.1	C12B—C13B—H13D	114.7
C8—C9—H9B	109.1	H13C—C13B—H13D	111.8
C10—C9—H9B	109.1	C13B—C14B—H14D	109.5
H9A—C9—H9B	107.9	C13B—C14B—H14E	109.5
O3—C10—O2	124.3 (3)	H14D—C14B—H14E	109.5
O3—C10—C9	124.9 (3)	C13B—C14B—H14F	109.5
O2—C10—C9	110.8 (2)	H14D—C14B—H14F	109.5
O2—C11A—C12A	107.2 (8)	H14E—C14B—H14F	109.5
O2—C11A—H11A	110.3	S—C15—H15A	109.5
C12A—C11A—H11A	110.3	S—C15—H15B	109.5
O2—C11A—H11B	110.3	H15A—C15—H15B	109.5
C12A—C11A—H11B	110.3	S—C15—H15C	109.5
H11A—C11A—H11B	108.5	H15A—C15—H15C	109.5
C11A—C12A—C13A	104.5 (6)	H15B—C15—H15C	109.5
			
O4—S—C1—C8	−136.3 (2)	C2—C1—C8—O1	−0.3 (3)
C15—S—C1—C8	114.3 (2)	S—C1—C8—O1	176.29 (16)
O4—S—C1—C2	39.5 (2)	C2—C1—C8—C9	175.7 (2)
C15—S—C1—C2	−69.9 (2)	S—C1—C8—C9	−7.8 (4)
C8—C1—C2—C3	−177.6 (3)	C7—O1—C8—C1	−0.3 (2)
S—C1—C2—C3	6.1 (4)	C7—O1—C8—C9	−177.00 (19)
C8—C1—C2—C7	0.7 (3)	C1—C8—C9—C10	−73.0 (3)
S—C1—C2—C7	−175.63 (18)	O1—C8—C9—C10	102.8 (2)
C7—C2—C3—C4	1.4 (3)	C11A—O2—C10—O3	2.1 (6)
C1—C2—C3—C4	179.5 (2)	C11B—O2—C10—O3	−15.4 (11)
C2—C3—C4—C5	−0.3 (3)	C11A—O2—C10—C9	−176.9 (5)
C2—C3—C4—Br	−179.94 (16)	C11B—O2—C10—C9	165.7 (10)
C3—C4—C5—C6	−0.5 (4)	C8—C9—C10—O3	24.4 (4)
Br—C4—C5—C6	179.14 (18)	C8—C9—C10—O2	−156.7 (2)
C4—C5—C6—C7	0.1 (4)	C10—O2—C11A—C12A	174.3 (5)
C8—O1—C7—C6	−179.6 (2)	C11B—O2—C11A—C12A	−74 (4)
C8—O1—C7—C2	0.8 (2)	O2—C11A—C12A—C13A	−78.9 (8)
C5—C6—C7—O1	−178.5 (2)	C11A—C12A—C13A—C14A	−172.1 (9)
C5—C6—C7—C2	1.1 (4)	C10—O2—C11B—C12B	128.1 (15)
C3—C2—C7—O1	177.69 (19)	C11A—O2—C11B—C12B	51 (3)
C1—C2—C7—O1	−0.9 (2)	O2—C11B—C12B—C13B	69 (3)
C3—C2—C7—C6	−1.9 (3)	C11B—C12B—C13B—C14B	−171 (3)
C1—C2—C7—C6	179.5 (2)		

### Hydrogen-bond geometry (Å, °)

**Table:**

*D*—H···*A*	*D*—H	H···*A*	*D*···*A*	*D*—H···*A*
C12A—H12A···Cg^i^	0.97	2.78	3.698 (5)	158
C5—H5···O3^ii^	0.93	2.55	3.405 (3)	153
C9—H9B···O4^iii^	0.97	2.30	3.248 (3)	167

Symmetry codes: (i) *x*+1, *y*+1, *z*; (ii) −*x*, −*y*+1, −*z*; (iii) −*x*+1, −*y*+1, −*z*+1.
